# Use of Electronic Cigarettes Among Cannabis-Naive Adolescents and Its Association With Future Cannabis Use

**DOI:** 10.1001/jamanetworkopen.2022.23277

**Published:** 2022-07-22

**Authors:** Ruoyan Sun, David Mendez, Kenneth E. Warner

**Affiliations:** 1 Department of Health Policy and Organization, School of Public Health, University of Alabama at Birmingham; 2 Department of Health Management and Policy, School of Public Health, University of Michigan, Ann Arbor

## Abstract

**Importance:**

Electronic cigarette (e-cigarette) use has been reported to increase the likelihood of future cigarette smoking among adolescents. The prospective association between e-cigarette use and cannabis use has been less clear, especially in recent years.

**Objective:**

To examine the association between e-cigarette use among cannabis-naive adolescents and cannabis use 1 year later.

**Design, Setting, and Participants:**

The Population Assessment of Tobacco and Health (PATH) Study, a nationally representative cohort study, uses a 4-stage, stratified probability sample design to select participants aged 12 years or older from the US civilian, noninstitutionalized population. This study sample included 9828 cannabis-naive adolescents at the baseline survey who participated in both wave 4.5 (2017-2018) and wave 5 (2018-2019) of PATH.

**Exposures:**

e-Cigarette use, assessed by ever use, past 12-month use, and past 30-day use.

**Main Outcomes and Measures:**

Cannabis use in wave 5, assessed by past 12-month and past 30-day use. Multivariable logistic regressions assessed the association between e-cigarette use and cannabis use 1 year later. Results were weighted to produce nationally representative findings.

**Results:**

Of the 9828 adolescents included in the analysis, 5361 (57.3%) were aged 12 to 14 years, 5056 (50.7%) were male, and 4481 (53.0%) were non-Hispanic White. After adjustment for sociodemographic characteristics, environmental factors, other substance use, and sensation seeking, e-cigarette use among cannabis-naive adolescents was associated with increased likelihoods of both self-reported past 12-month and past 30-day cannabis use 1 year later. The adjusted relative risks (aRRs) of subsequent past 12-month cannabis use with ever use of e-cigarettes was 2.57 (95% CI, 2.04-3.09), with past 12-month use of e-cigarettes was 2.62 (95% CI, 2.10-3.15), and with past 30-day use of e-cigarettes was 2.18 (95% CI, 1.50-2.85). The aRRs of subsequent past 30-day cannabis use with ever use of e-cigarettes was 3.20 (95% CI, 2.10-4.31), with past 12-month use of e-cigarettes was 3.40 (95% CI, 2.17-4.63), and with past 30-day use of e-cigarettes was 2.96 (95% CI, 1.52-4.40).

**Conclusions and Relevance:**

This cohort study’s findings suggest a strong association between adolescent e-cigarette use and subsequent cannabis use. However, despite the strong association at the individual level, e-cigarette use seems to have had a minimal association with the prevalence of youth cannabis use at the population level.

## Key Points

**Question:**

Is electronic cigarette use among cannabis-naive adolescents in the US associated with increased likelihood of future cannabis use?

**Findings:**

With the use of longitudinal data on a nationally representative cohort of 9828 youths from 2017 to 2019, this cohort study found that cannabis-naive adolescents who have used electronic cigarettes are significantly more likely to report cannabis use 1 year later compared with those who have not used electronic cigarettes.

**Meaning:**

The findings of this cohort study suggest that adolescent electronic cigarette use was associated with an increased likelihood of future cannabis use, but the overall association of electronic cigarette use with youth cannabis use at the population level is likely quite small.

## Teaser

This cohort study uses data from the Population Assessment of Tobacco and Health Study to examine the association between e-cigarette use among cannabis-naive adolescents and cannabis use 1 year later.

## Introduction

Since 2014, electronic cigarettes (e-cigarettes) have become the most commonly used nicotine-containing product among US middle school and high school students.^1,2^ According to the National Youth Tobacco Survey, the prevalence of past 30-day e-cigarette use increased from 11.7% to 27.5% among high school students from 2017 to 2019.^3,4^ For middle school students, the prevalence of past 30-day e-cigarette use increased from 3.3% to 10.5% from 2017 to 2019. e-Cigarette use decreased significantly among middle school and high school students in 2020 and 2021; 11.3% of high school students and 2.8% of middle school students reported past 30-day e-cigarette use in 2021.^5,6^

The popularity of e-cigarettes has raised concern owing to their potential to serve as a gateway to smoking cigarettes.^7,8^ Many studies have found that youths who use e-cigarettes have higher odds of future cigarette smoking compared with those who do not use e-cigarettes. A systematic review of 17 studies comprising youths and young adults found strong evidence for a significant association between e-cigarette use and later smoking, with an odds ratio (OR) of 4.59 (95% CI, 3.60-5.85).^9^ However, the positive association may disappear with a comprehensive selection of control variables.^10^

Although most studies have focused on the association between e-cigarette use and smoking cigarettes, similar concerns have been raised for the use of e-cigarettes as a gateway to cannabis use.^11,12^ Vaping (use of e-cigarettes and similar devices) involves heating liquid, oil, or plant material to a temperature that releases an aerosolized mixture of water vapor and active ingredients, such as nicotine in e-cigarettes and tetrahydrocannabinol (THC) in cannabis. The availability of these vaping devices makes it easier for adolescents to try cannabis. The National Academies of Sciences, Engineering, and Medicine reported evidence that cannabis use among youths is correlated with impaired cognitive abilities and life achievement, along with increased rates of mental health disorders, such as depression and anxiety.^13^

A growing body of literature has found that youths who use e-cigarettes are more likely to initiate cannabis use than their peers who do not use e-cigarettes.^14,15,16,17,18,19,20,21^ A meta-analysis from Chadi et al^21^ reported that the odds of cannabis use increased significantly among youths who used e-cigarettes, both in longitudinal studies (adjusted OR [aOR], 2.43 [95% CI, 1.51-3.90]) and in cross-sectional studies (aOR, 3.70 [95% CI, 2.76-4.96]).

All of the longitudinal studies assessing the association between e-cigarette use and cannabis use among US adolescents examined data from 2013 to 2017.^13,16,17,18,19^ Little is known about whether this positive association has persisted more recently, especially from 2017 to 2019 when the prevalence of youth e-cigarette use rapidly increased. To address this gap, we analyzed longitudinal data from the 2 most recent waves of the Population Assessment of Tobacco and Health (PATH) Study (wave 4.5 [2017-2018] and wave 5 [2018-2019]). We also examined multiple measures of e-cigarette use and cannabis use. We assessed e-cigarette use using ever, past 12-month, and past 30-day use, with cannabis use examined via past 12-month and past 30-day use. Thus, we investigated the association between baseline e-cigarette use and cannabis use 1 year later among never cannabis users at baseline, controlling for multiple independent risk factors, including sociodemographic characteristics, environmental factors, other substance use, and sensation seeking.

## Methods

The PATH Study is a nationally representative, longitudinal cohort study of youths and adults in the US. The study uses a 4-stage, stratified probability sample design to select youths and adults from the US civilian, noninstitutionalized population. Surveys were conducted via audio computer-assisted self-interviews and computer-assisted personal interviews to collect self-reported data on tobacco use and related health behaviors. A full description of the PATH Study design and methods is available elsewhere.^22^ Our sample consisted of youths aged 12 to 17 years who participated in both wave 4.5 (2017-2018) and wave 5 (2018-2019) of the PATH Study, including those who turned 18 years of age in wave 5 and were therefore included in the adult survey. The weighted response rate was 83.5% for wave 5 youths and 88.0% for wave 5 adults.^23^ Our cohort study comprises 9828 youths who had never used cannabis (cannabis-naive) by wave 4.5. See the eFigure in the Supplement for a flowchart of sample construction. Adolescents were recruited for the PATH Study after written consent was given by the parents. The University of Alabama at Birmingham institutional review board exempted this study from review because it used deidentified data. This study followed the Strengthening the Reporting of Observational Studies in Epidemiology (STROBE) reporting guideline.

### Measures

The independent variable of interest was self-reported e-cigarette use by wave 4.5, assessed by 3 different measures: ever use of e-cigarettes, past 12-month use of e-cigarettes, and past 30-day use of e-cigarettes. The main outcome measures were past 12-month use and past 30-day cannabis use in wave 5. Past 12-month use of cannabis was defined by an affirmative answer to any of the following 4 questions in the PATH Study: “In the past 12 months, have you used marijuana, hash, THC, grass, pot, or weed?” “In the past 12 months, have you smoked part or all of a traditional cigar, cigarillo, or filtered cigar with marijuana in it?” “Have you ever smoked marijuana in a hookah?” “Have you ever used marijuana, marijuana concentrates, marijuana waxes, THC, or hash oils in an electronic product such as an e-cigarette, vape, mod, personal vaporizer, e-hookah, or hookah pen?” Past 30-day cannabis use was defined by responses to the following 2 questions: “Have you used marijuana, hash, THC, grass, pot, or weed in the past 30 days?” “When was the last time you smoked a traditional cigar/cigarillo/filtered cigar as a blunt, even 1 or 2 puffs?” We converted the latter question into a binary measure of 1 to 30 days or no use. Participants reporting “yes” to the first 30-day question or 1 to 30 days in response to the second question were categorized as past 30-day cannabis users. Those who answered “don’t know” or “refused” were considered missing for that question.

#### Sociodemographic Characteristics

Consistent with previous studies, we included basic sociodemographic variables as covariates. Sociodemographic variables included age (12-14 years vs 15-17 years), sex (male vs female), race and ethnicity (Hispanic, non-Hispanic Black, non-Hispanic White, and non-Hispanic other [includes American Indian or Alaska Native, Asian Indian, Chinese, Filipino, Japanese, Korean, Vietnamese, other Asian, Native Hawaiian, Guamanian or Chamorro, Samoan, and Other Pacific Islander]), highest parental educational level (≤high school or GED [General Educational Development certification], some college, and ≥college), household income (<$50 000, $50 000-$100 000, and >$100 000), and school grades (<mostly B’s vs ≥mostly B’s). Questions on highest parental educational level, household income, and school grades were answered by the parent, whereas the other questions were answered by the adolescent participant.

#### Environmental Factors

Family tobacco use (0 vs 1) was evaluated by asking if anyone living with the respondent used cigarettes, smokeless tobacco, cigars, cigarillos, filtered cigars, or any other form of tobacco. Peer tobacco use (0 vs 1) was scored 1 if participants reported any positive number to questions asking, “How many of your best friends use [tobacco product]?” Tobacco products included were cigarettes, e-cigarettes, cigarillos, snus, and smokeless tobacco.

#### Other Substance Use

Ever use of tobacco products other than e-cigarettes (0 vs 1) was defined by any positive response to questions asking about ever use of cigarette, cigar, pipe, hookah, snus, smokeless tobacco, bidi, kretek, or dissolvable tobacco. Past 12-month use of alcohol (0 vs 1) was assessed by the question, “In the past 12 months, have you used any alcohol?” Participants who answered “yes” to the question, “Have you ever used any of the following prescription drugs (Ritalin or Adderall, painkillers, sedatives, or tranquilizers) that were not prescribed for you or that you took only for the experience or feeling they caused?” were considered as ever nonmedical users of prescription drugs.

#### Sensation Seeking

We assessed sensation seeking using 3 questions measured on a 5-point scale (where 1 indicates strongly disagree and 5 indicates strongly agree), modified from the Brief Sensation Seeking Scale.^24^ The sensation seeking score was calculated as the mean response to these 3 questions, which asked about respondents’ affinity for frightening things, new and exciting experiences, and exciting and unpredictable friends. The score was treated as a continuous variable.^24,25^ Questions on sensation seeking were asked only once when the participants first joined the PATH Study.

### Statistical Analysis

We conducted the statistical analysis using Stata, version 17 (StataCorp LLC), with the Fay method of balanced repeated replication to estimate variance. Stata’s svy command was used to incorporate survey weights. Multivariable logistic regressions were performed to examine the association between e-cigarette use at baseline (wave 4.5) and cannabis use 1 year later (wave 5), controlling for the variables identified. All *P* values were from 2-sided tests, and results were deemed statistically significant at *P* < .05.

We report our main results as adjusted relative risks (aRRs) and adjusted risk differences (aRDs), calculated using Stata’s margins command. Other studies have reported the association in aORs.^14,16,17,18,19^ However, ORs are commonly misinterpreted as relative risks^26,27^ and would produce biased estimates of relative risks given the high prevalence of our outcome in wave 5.^28,29^ This bias is demonstrated by comparing our main results in aRRs with aORs in eTable 1 in the Supplement.

#### Missing Data

The variable with the most missing data was sensation seeking (14.7%), while other variables had few missing values (<5%). Our complete-case analysis excluded participants with missing data.

#### Sensitivity Analyses

To examine the robustness of the association between e-cigarette use and subsequent cannabis use, we conducted several sensitivity analyses: (1) adding psychological measures of internalizing and externalizing problems,^30,31^ (2) removing sensation seeking owing to a large number of cases with missing values, and (3) considering participants answering “don’t know” or “refused” as users or nonusers of that product (instead of missing).

## Results

Results were weighted to produce nationally representative findings. Of the 9828 adolescents included in the analysis, 5361 (57.3% [95% CI, 56.8%-57.9%]) were aged 12 to 14 years, 5056 (50.7% [95% CI, 50.3%-51.2%]) were male, and 4481 (53.0% [95% CI, 52.4%-53.5%]) were non-Hispanic White (Table 1). Highest parental educational level was high school or GED or less for 2824 individuals (25.6% [95% CI, 24.5%-26.8%]), some college for 2822 individuals (28.2% [95% CI, 26.7%-29.6%]), and college or higher for 4088 individuals (46.2% [95% CI, 44.6%-47.9%]). Annual household income was less than $50 000 for 4087 participants (39.2% [95% CI, 38.0%-40.5%]), between $50 000 and $100 000 for 2393 participants (26.4% [95% CI, 25.2%-27.5%]), and more than $100 000 for 2894 participants (34.3% [95% CI, 32.8%-36.0%]). A total of 2472 participants (24.2% [95% CI, 23.3%-25.2%]) reported grades less than mostly B’s, and a total of 7175 participants (75.8% [95% CI, 74.8%-76.7%]) reported mostly B’s or higher. A total of 2649 participants (27.4% [95% CI, 26.2%-28.7%]) had family members who used tobacco, and a total of 2859 participants (29.2% [95% CI, 28.0%-30.4%]) had best friends who used tobacco. A small fraction (465 [4.8%; 95% CI, 4.3%-5.4%]) had ever used any tobacco product other than e-cigarettes, but 1824 participants (19.2% [95% CI, 18.0%-20.4%]) reported past 12-month alcohol use. A total of 1272 participants (12.6% [95% CI, 11.9%-13.4%]) reported ever nonmedical use of prescription drugs. The mean (SE) sensation seeking score was 2.43 (0.01).

**Table 1.** Sample Characteristics of Baseline Never Cannabis Users, Overall and Stratified by Ever e-Cigarette Use Status^a^

Characteristic of wave 4.5 never cannabis users	No. (weighted %) [95% CI]			*P* value^b^
	Total (N = 9828)	e-Cigarette users		
		Never (n = 9027)	Ever (n = 757)	
Age, y				
12-14	5361 (57.3) [56.8-57.9]	5122 (59.8) [59.2-60.4]	220 (28.8) [25.2-32.7]	<.001
15-17	4467 (42.7) [42.1-43.2]	3905 (40.2) [39.6-40.8]	537 (71.2) [67.3-74.8]	
Sex				
Male	5056 (50.7) [50.3-51.2]	4629 (50.6) [50.0-51.1]	406 (52.9) [48.4-57.4]	.30
Female	4734 (49.3) [48.8-49.7]	4366 (49.4) [48.9-50.0]	347 (47.1) [42.6-51.6]	
Race and ethnicity				
Hispanic	2787 (23.8) [23.4-24.2]	2586 (24.1) [23.6-24.6]	191 (19.9) [16.3-24.1]	<.001
Non-Hispanic Black	1178 (12.5) [12.1-13.0]	1132 (13.1) [12.7-13.6]	37 (4.9) [3.5-6.9]	
Non-Hispanic White	4481 (53.0) [52.4-53.5]	4028 (51.8) [51.2-52.3]	442 (67.7) [63.0-72.1]	
Non-Hispanic other^c^	921 (10.7) [10.4-11.1]	863 (11.0) [10.7-11.4]	57 (7.4) [5.4-10.3]	
Highest parental educational level				
≤High school or GED	2824 (25.6) [24.5-26.8]	2584 (25.5) [24.3-26.7]	222 (26.2) [22.4-30.4]	.34
Some college	2822 (28.2) [26.7-29.6]	2612 (28.4) [26.9-30.0]	198 (25.5) [22.0-29.4]	
≥College	4088 (46.2) [44.6-47.9]	3743 (46.1) [44.4-47.8]	331 (48.3) [43.1-53.5]	
Household income, $				
<50 000	4087 (39.2) [38.0-40.5]	3760 (39.3) [38.1-40.6]	301 (37.2) [32.3-42.4]	.60
50 000-100 000	2393 (26.4) [25.2-27.5]	2198 (26.4) [25.2-27.7]	188 (26.5) [22.6-30.9]	
>100 000	2894 (34.3) [32.8-36.0]	2646 (34.3) [32.7-36.0]	240 (36.3) [31.3-41.5]	
School grades				
<Mostly B’s	2472 (24.2) [23.3-25.2]	2192 (23.4) [22.5-24.4]	265 (33.2) [29.2-37.6]	<.001
≥Mostly B’s	7175 (75.8) [74.8-76.7]	6663 (76.6) [75.6-77.5]	484 (66.8) [62.5-70.8]	
Family tobacco use				
Yes	2649 (27.4) [26.2-28.7]	2330 (26.2) [24.9-27.5]	307 (42.4) [38.1-46.8]	<.001
No	7087 (72.6) [71.3-73.8]	6612 (73.8) [72.5-75.1]	443 (57.6) [53.2-61.9]	
Peer tobacco use^d^				
Yes	2859 (29.2) [28.0-30.4]	2325 (25.7) [24.5-26.9]	528 (71.8) [68.1-75.2]	<.001
No	6914 (70.8) [69.6-72.0]	6651 (74.3) [73.1-75.5]	226 (28.2) [24.8-31.9]	
Ever used tobacco products other than e-cigarettes^e^				
Yes	465 (4.8) [4.3-5.4]	272 (3.1) [2.7-3.5]	193 (25.8) [21.7-30.5]	<.001
No	9004 (95.2) [94.7-95.7]	8440 (96.9) [96.5-97.3]	534 (74.2) [69.6-78.3]	
Used alcohol in past 12 mo				
Yes	1824 (19.2) [18.0-20.4]	1495 (17.1) [15.9-18.3]	325 (44.6) [40.0-49.2]	<.001
No	7977 (80.8) [79.6-82.0]	7509 (82.9) [81.7-84.1]	430 (55.5) [50.8-60.0]	
Ever nonmedical prescription drug use^f^				
Yes	1272 (12.6) [11.9-13.4]	1097 (11.9) [11.1-12.7]	165 (20.7) [17.7-24.0]	<.001
No	8337 (87.4) [86.6-88.1]	7735 (88.1) [87.3-88.9]	571 (79.3) [76.0-82.3]	
Sensation seeking score, mean (SE)^g^	2.43 (0.01)	2.40 (0.01)	2.78 (0.05)	
No.	8314	7659	632	<.001

Abbreviation: GED, General Educational Development certification.

^a^ Due to missing data, sample sizes across variables may not add up to the total sample sizes.

^b^ Pearson χ^2^ test was performed to compare the distribution of characteristics by e-cigarette use status at wave 4.5.

^c^ Includes American Indian or Alaska Native, Asian Indian, Chinese, Filipino, Japanese, Korean, Vietnamese, other Asian, Native Hawaiian, Guamanian or Chamorro, Samoan, and Other Pacific Islander.

^d^ Peer tobacco use was assessed via best friends’ use of cigarettes, e-cigarettes, cigarillos, snus, or smokeless tobacco.

^e^ Tobacco products other than e-cigarettes include cigarettes, cigars, pipe, hookah, snus, smokeless tobacco, bidis, kreteks, and dissolvable tobacco.

^f^ Prescription drugs include Ritalin, Adderall, painkillers, sedatives, or tranquilizers.

^g^ A score from 1 to 5 that aggregated three 5-point Likert-type measures of affinity for frightening things, new and exciting experiences, and exciting and unpredictable friends (where 1 indicates strongly disagree and 5 indicates strongly agree).

We found significant differences between ever users and never users of e-cigarettes. Those reporting ever e-cigarette use were more likely than those reporting never e-cigarette use to be older (15-17 y, 71.2% [95% CI, 67.3%-74.8%] vs 40.2% [95% CI, 39.6%-40.8%]) and non-Hispanic White (67.7% [95% CI, 63.0%-72.1%] vs 51.8% [95% CI, 51.2%-52.3%]) and to have lower school grades (less than mostly B’s, 33.2% [95% CI, 29.2%-37.6%] vs 23.4% [95% CI, 22.5%-24.4%]), family members who used any tobacco product (42.4% [95% CI, 38.1%-46.8%] vs 26.2% [95% CI, 24.9%-27.5%]), and best friends who used any tobacco product (71.8% [95% CI, 68.1%-75.2%] vs 25.7% [95% CI, 24.5%-26.9%]) (Table 1). They were also more likely to report ever use of tobacco products other than e-cigarettes (25.8% [95% CI, 21.7%-30.5%] vs 3.1% [95% CI, 2.7%-3.5%]), past 12-month alcohol use (44.6% [95% CI, 40.0%-49.2%] vs 17.1% [95% CI, 15.9%-18.3%]), ever nonmedical use of prescription drugs (20.7% [95% CI, 17.7%-24.0%] vs 11.9% [95% CI, 11.1%-12.7%]), and higher mean (SE) sensation seeking scores (2.78 [0.05] vs 2.40 [0.01]).

In Table 2, we present the proportion of baseline never cannabis users who reported subsequent past 12-month and past 30-day cannabis use by selected characteristics. Among baseline cannabis-naive adolescents consisting of both users and nonusers of e-cigarettes, 1 year later, 10.7% (95% CI, 9.9%-11.6%) reported past 12-month cannabis use, and 4.7% (95% CI, 4.2%-5.2%) reported past 30-day cannabis use. In the past 12 months, 38.8% (95% CI, 34.6%-43.3%) of ever e-cigarette users initiated cannabis use compared with 8.3% (95% CI, 7.6%-9.1%) of never e-cigarette users. In the past 30 days, 19.3% (95% CI, 16.0%-23.0%) of ever e-cigarette users used cannabis compared with 3.4% (95% CI, 3.0%-3.9%) of never e-cigarette users. Baseline past 12-month and past 30-day e-cigarette use were also factors significantly associated with subsequent past 12-month and past 30-day cannabis use. In addition, age, school grades, family tobacco use, peer tobacco use, ever use of tobacco products other than e-cigarettes, past 12-month use of alcohol, and ever nonmedical use of prescription drugs were significantly associated with future cannabis use.

**Table 2.** Past 12-Month and Past 30-Day Cannabis Use at Wave 5 Among Baseline Never Cannabis Users, by Sample Characteristics

Wave 4.5 never cannabis users	Wave 5			
	Past 12-mo cannabis use		Past 30-d cannabis use	
	No. (weighted %) [95% CI]^a^	*P* value^b^	No. (weighted %) [95% CI]^a^	*P* value^b^
Total	917 (10.7) [9.9-11.6]		393 (4.7) [4.2-5.2]	
Ever used e-cigarettes				
Yes	244 (38.8) [34.6-43.3]	<.001	120 (19.3) [16.0-23.0]	<.001
No	672 (8.3) [7.6-9.1]		272 (3.4) [3.0-3.9]	
Used e-cigarettes in past 12 mo				
Yes	198 (42.3) [37.5-47.3]	<.001	102 (21.9) [17.7-26.8]	<.001
No	718 (8.7) [8.0-9.5]		290 (3.6) [3.2-4.0]	
Used e-cigarettes in past 30 d				
Yes	90 (43.4) [36.3-50.9]	<.001	47 (24.8) [18.4-32.5]	<.001
No	826 (9.9) [9.1-10.7]		345 (4.1) [3.7-4.6]	
Age, y				
12-14	346 (7.1) [6.2-8.0]	<.001	137 (2.9) [2.4-3.6]	<.001
15-17	571 (15.7) [14.3-17.1]		256 (7.1) [6.2-8.0]	
Sex				
Male	445 (10.1) [9.1-11.2]	.12	191 (4.5) [3.8-5.2]	.39
Female	472 (11.3) [10.2-12.6]		202 (4.9) [4.2-5.6]	
Race and ethnicity				
Hispanic	281 (11.1) [9.6-12.7]	.06	116 (4.4) [3.6-5.4]	.68
Non-Hispanic Black	136 (12.6) [10.7-14.8]		52 (5.2) [3.9-6.8]	
Non-Hispanic White	420 (10.5) [9.5-11.7]		190 (4.8) [4.1-5.6]	
Non-Hispanic other^c^	80 (8.6) [6.8-10.8]		35 (4.0) [2.8-5.8]	
Highest parental educational level				
≤High school or GED	283 (12.0) [10.5-13.8]	.11	128 (5.4) [4.6-6.3]	.17
Some college	280 (10.8) [9.5-12.2]		106 (4.2) [3.4-5.0]	
≥College	354 (10.0) [8.8-11.3]		159 (4.6) [3.9-5.5]	
Household income, $				
<50 000	411 (11.3) [10.1-12.6]	.41	164 (4.6) [3.9-5.4]	.94
50 000-100 000	238 (10.7) [9.4-12.3]		107 (4.6) [3.8-5.7]	
>100 000	268 (10.1) [8.8-11.5]		122 (4.8) [4.0-5.8]	
School grades				
<Mostly B’s	305 (14.8) [12.8-17.0]	<.001	118 (5.8) [4.6-7.3]	.03
≥Mostly B’s	612 (9.5) [8.8-10.3]		275 (4.3) [3.8-4.9]	
Family tobacco use				
Yes	346 (15.1) [13.5-16.9]	<.001	150 (6.5) [5.6-7.5]	<.001
No	571 (9.0) [8.3-9.9]		243 (4.0) [3.5-4.6]	
Peer tobacco use^d^				
Yes	500 (20.5) [18.7-22.4]	<.001	219 (9.0) [7.8-10.4]	<.001
No	417 (6.7) [6.0-7.4]		174 (2.9) [2.4-3.4]	
Ever used tobacco products other than e-cigarettes^e^				
Yes	119 (30.7) [25.8-36.2]	<.001	51 (13.7) [10.5-17.6]	<.001
No	798 (9.7) [9.0-10.5]		342 (4.2) [3.8-4.7]	
Used alcohol in past 12 mo				
Yes	365 (22.3) [20.0-24.8]	<.001	152 (9.0) [7.5-10.8]	<.001
No	552 (7.8) [7.1-8.6]		241 (3.6) [3.1-4.1]	
Ever nonmedical prescription drug use^f^				
Yes	188 (16.9) [14.8-19.3]	<.001	78 (7.4) [6.2-8.9]	<.001
No	729 (9.8) [9.0-10.6]		315 (4.3) [3.8-4.8]	
Sensation seeking score, mean (SE)^g^	2.77 (0.04)		2.82 (0.07)	NA

Abbreviations: GED, General Educational Development certification; NA, not applicable.

^a^ Excluded observations with missing data for wave 5 cannabis use and wave 4.5 covariates except for baseline e-cigarette use owing to different missing patterns among e-cigarette use variables (ever, past 12 months, and past 30 days).

^b^ Pearson χ^2^ test was performed to compare the distribution of cannabis use status at wave 5 by sample characteristics.

^c^ Includes American Indian or Alaska Native, Asian Indian, Chinese, Filipino, Japanese, Korean, Vietnamese, other Asian, Native Hawaiian, Guamanian or Chamorro, Samoan, and Other Pacific Islander.

^d^ Peer tobacco use was assessed via best friends’ use of cigarettes, e-cigarettes, cigarillos, snus, or smokeless tobacco.

^e^ Tobacco products other than e-cigarettes include cigarettes, cigars, pipe, hookah, snus, smokeless tobacco, bidis, kreteks, and dissolvable tobacco.

^f^ Prescription drugs include Ritalin, Adderall, painkillers, sedatives, or tranquilizers.

^g^ A score from 1 to 5 that aggregated three 5-point Likert-type measures of affinity for frightening things, new and exciting experiences, and exciting and unpredictable friends (where 1 indicates strongly disagree and 5 indicates strongly agree).

Table 3 shows the association of baseline e-cigarette use and future 12-month cannabis use after adjusting for all study covariates. Ever e-cigarette use at baseline was associated with a 13.93–percentage point increase (95% CI, 9.83-18.04 percentage points) in reported past 12-month cannabis use 1 year later, from 8.90% (95% CI, 8.05%-9.75%) for never e-cigarette users to 22.84% (95% CI, 18.92%-26.75%) for ever e-cigarette users. Past 12-month e-cigarette use was associated with a 14.89–percentage point increase (95% CI, 10.52-19.26 percentage points) in past 12-month cannabis use. Past 30-day e-cigarette use was associated with an increase of 11.86 percentage points (95% CI, 5.34-18.38 percentage points) in past 12-month cannabis use. The resulting aRR for 12-month cannabis use within the next year was 2.57 (95% CI, 2.04-3.09) for ever e-cigarette use at baseline, 2.62 (95% CI, 2.10-3.15) for past 12-month e-cigarette use at baseline, and 2.18 (95% CI, 1.50-2.85) for past 30-day e-cigarette use at baseline.

**Table 3.** Association Between Baseline e-Cigarette Use and Subsequent Past 12-Month Cannabis Use Among Baseline Never Cannabis Users

Wave 4.5 e-cigarette use	Past 12-mo cannabis use in wave 5				
	aRR (95% CI)^a^	aRD (95% CI), percentage points^b^	Risk without e-cigarette use, % (95% CI)	Risk with e-cigarette use, % (95% CI)	Sample size^c^
Ever e-cigarette use	2.57 (2.04-3.09)	13.93 (9.83-18.04)	8.90 (8.05-9.75)	22.84 (18.92-26.75)	7011
Past 12-mo e-cigarette use	2.62 (2.10-3.15)	14.89 (10.52-19.26)	9.17 (8.34-10.00)	24.06 (19.84-28.28)	7018
Past 30-d e-cigarette use	2.18 (1.50-2.85)	11.86 (5.34-18.38)	10.07 (9.23-10.91)	21.93 (15.62-28.24)	7017

Abbreviations: aRD, adjusted risk difference; aRR, adjusted relative risk.

^a^ Adjusted for all study covariates: age, sex, race and ethnicity, highest parental educational level, household income, school grades, family tobacco use, peer tobacco use, ever tobacco product use (excluding e-cigarettes), past 12-month alcohol use, ever nonmedical use of prescription drugs, and sensation seeking.

^b^ Adjusted for all study covariates.

^c^ Effective sample size; participants with missing data were excluded.

Table 4 presents the association of e-cigarette use (wave 4.5) and future 30-day cannabis use (wave 5), adjusted for all study covariates. Ever e-cigarette use was associated with an increase in cannabis use from 3.61% (95% CI, 3.06%-4.16%) to 11.57% (95% CI, 8.24%-14.89%), an increase of 7.96 percentage points (95% CI, 4.49-11.42 percentage points). Past 12-month use of e-cigarettes was associated with an increase of 8.94 percentage points (95% CI, 4.77-13.11 percentage points), and past 30-day use of e-cigarettes was associated with an increase of 8.29 percentage points (95% CI, 2.30-14.29 percentage points). The aRR for future past 30-day cannabis use was 3.20 (95% CI, 2.10-4.31) for ever e-cigarette use at baseline, 3.40 (95% CI, 2.17-4.63) for past 12-month e-cigarette use at baseline, and 2.96 (95% CI, 1.52-4.40) for past 30-day e-cigarette use at baseline. Complete regression results can be found in eTables 2 to 4 in the Supplement.

**Table 4.** Association Between Baseline e-Cigarette Use and Subsequent Past 30-Day Cannabis Use Among Baseline Never Cannabis Users

Wave 4.5 e-cigarette use	Past 30-d cannabis use in wave 5					
	aRR (95% CI)^a^	aRD (95% CI), percentage points^b^	Risk without e-cigarette use, % (95% CI)	Risk with e-cigarette use, % (95% CI)	Sample size^c^
Ever e-cigarette use	3.20 (2.10-4.31)	7.96 (4.49-11.42)	3.61 (3.06-4.16)	11.57 (8.24-14.89)	7032
Past 12-mo e-cigarette use	3.40 (2.17-4.63)	8.94 (4.77-13.11)	3.72 (3.21-4.24)	12.66 (8.59-16.74)	7039
Past 30-d e-cigarette use	2.96 (1.52-4.40)	8.29 (2.30-14.29)	4.23 (3.73-4.73)	12.52 (6.57-18.48)	7038

Abbreviations: aRD, adjusted risk difference; aRR, adjusted relative risk.

^a^ Adjusted for all study covariates: age, sex, race and ethnicity, highest parental educational level, household income, school grades, family tobacco use, peer tobacco use, ever tobacco product use (excluding e-cigarettes), past 12-month alcohol use, ever nonmedical use of prescription drugs, and sensation seeking.

^b^ Adjusted for all study covariates.

^c^ Effective sample size; participants with missing data were excluded.

The results of all of the sensitivity analyses are similar to those in Table 3 and Table 4. See eTable 5 in the Supplement for definitions of internalizing and externalizing problems and eTables 6 to 8 in the Supplement for sensitivity analysis results. Given the popularity of cannabis vaping, we also examined the prospective association between e-cigarette use and past 12-month cannabis vaping. The aRRs were close to our main results (eTable 9 in the Supplement).

## Discussion

Consistent with previous literature using data from 2013 to 2017,^14,15,16,17,18,19,20^ we found a significant and robust association between baseline e-cigarette use among cannabis-naive youth and subsequent cannabis use. We add to this literature by using more recent nationally representative data from 2017 to 2019, a period with substantial increases in vaping prevalence. For example, among high school students, past 30-day use of e-cigarettes increased from 4.5% in 2013 to 11.7% in 2017 and then increased to 27.5% in 2019.^3,4,32^ We present our results as aRRs and aRDs rather than the aORs used in most previous studies. (However, 2 of these studies did present results in aRRs.^15,20^) As observed, aRRs and aRDs provide a more readily comprehensible assessment of the association between e-cigarette use and subsequent cannabis use.^26,27^ Unlike most previous studies, we examined the association via multiple measures of e-cigarette use (ever, past 12 months, and past 30 days) and cannabis use (past 12 months and past 30 days). All comparisons yielded similar results.

There are a few possible explanations for the prospective association between e-cigarette use and cannabis use. Even though we controlled for sensation seeking and some risky behaviors, e-cigarette use may be a marker for other risk-taking behaviors that are also associated with cannabis use. e-Cigarette users may be more likely to befriend peers who engage in other risky behaviors, such as cannabis use. Peer pressure has been identified by many studies as a risk factor for cannabis use.^33,34^ Another possible reason for this association is the increased prevalence of vaping as a means of using cannabis. The prevalence of past 12-month cannabis vaping among 12th-grade students increased from 9.5% in 2017 to 22.1% in 2020.^35^ More than half of current e-cigarette users aged 15 to 24 years reported cannabis vaping.^36^ e-Cigarette use could increase adolescents’ likelihood to vape cannabis because the same vaping devices can be used for both products. eTable 9 in the Supplement shows the prospective association between e-cigarette use and past 12-month cannabis vaping. The aRRs were close to our main findings, suggesting a limited difference across modes of cannabis use. Similar findings were also reported by another study.^37^ The lack of difference is likely because most cannabis users consume cannabis via multiple modes, including vaping.

The association between e-cigarette use and cannabis use at the individual level appears inconsistent with their use at the population level. Although there is clearly a strong association between e-cigarette use and subsequent cannabis use at the individual level,^14,15,16,17,18,19,20^ the prevalence of adolescent cannabis use at the population level has remained relatively stable from 1995 to 2020 among adolescents in 8th, 10th, and 12th grade.^35^ There are some possible explanations for this discrepancy. First, despite the association between individuals’ e-cigarette use and subsequent cannabis use, the size of this population (ie, cannabis-naive adolescents who have tried e-cigarettes) is relatively small, resulting in minimal changes in cannabis use at the population level. For example, 7.8% (95% CI, 7.3%-8.3%) of cannabis-naive adolescents at wave 4.5 had ever used e-cigarettes. That is 6.3% (95% CI, 5.9%-6.8%) of the entire youth population. Even if the estimated association were completely causal, our calculations in eTable 10 in the Supplement demonstrate that the estimated change in cannabis use at the population level due to e-cigarette use is less than 1 percentage point. Another explanation is that subsequent cannabis use associated with e-cigarette use may not persist over time. Some adolescent e-cigarette users likely simply experiment with cannabis use without becoming established users.

### Limitations

This study has some limitations. One potentially important factor associated with cannabis use is the legalization of the recreational use of cannabis for adults in several states. Although it is still illegal for adolescents to purchase cannabis, cannabis legalization for adults may make it easier for adolescents to access cannabis. Owing to a lack of geographic information, our study was unable to control for state-level cannabis legalization. In 2019, 20.4% of all 12- to 17-year-old adolescents lived in states with licensed sales for recreational cannabis.^38^ However, with four-fifths of adolescents not living in states with recreational cannabis sales, we suspect that legalization of recreational cannabis is not associated with our results. Nevertheless, the effect of legalizing recreational cannabis use is a topic worthy of study.

Another limitation is the possibility of response bias in the PATH Study’s self-reported data, including e-cigarette use and cannabis use. However, Bachman et al^39^ found evidence supporting the validity of self-reported data. In addition, although all of the results in this study were weighted to produce nationally representative findings, the participants excluded from our analysis owing to missing data might have affected the generalizability of our findings. Finally, there is 1 limitation associated with the PATH Study survey questions regarding cannabis. Questions on past 30-day cannabis use did not explicitly ask about cannabis use through hookah or electronic products, unlike questions on past 12-month use. Because some respondents provided inconsistent answers to questions about past 12-month cannabis use and cannabis use via hookah or electronic products (“no” to past 12-month cannabis use but “yes” to cannabis use via hookah or electronic products), the measure of past 30-day cannabis use likely missed some participants who used cannabis through hookah or electronic products.

## Conclusions

Using PATH Study data from 2017 to 2019, this cohort study found that, among cannabis-naive adolescents, those who have used e-cigarettes were significantly more likely to use cannabis 1 year later compared with those who had not used e-cigarettes. However, despite this association, e-cigarette use seems to have had a minimal association with the overall prevalence of youth cannabis use. At the population level, adolescent use of cannabis has remained relatively stable for the past quarter century.

## Notes

Supplement. **eFigure.** Flowchart for Sample Construction **eTable 1.** Association Between Baseline e-Cigarette Use and Subsequent Cannabis Use (Past 12-Month and Past 30-Day) Among Baseline Never Cannabis Users, Comparing aRRs and aORs **eTable 2.** Association Between Baseline Ever e-Cigarette Use and Subsequent Cannabis Use (Past 12-Month and Past 30-Day) Among Baseline Never Cannabis Users **eTable 3.** Association Between Baseline Past 12-Month e-Cigarette Use and Subsequent Cannabis Use (Past 12-Month and Past 30-Day) Among Baseline Never Cannabis Users **eTable 4.** Association Between Baseline Past 30-Day e-Cigarette Use and Subsequent Cannabis Use (Past 12-Month and Past 30-Day) Among Baseline Never Cannabis Users **eTable 5.** Global Appraisal of Individual Needs – Short Screener (GAIN-SS) Items **eTable 6.** Association Between Baseline e-Cigarette Use and Subsequent Cannabis Use Among Baseline Never Cannabis Users, With Additional Measures of Internalizing and Externalizing Problems **eTable 7.** Association Between Baseline e-Cigarette Use and Subsequent Cannabis Use Among Baseline Never Cannabis Users, Without Sensation Seeking **eTable 8.** Association Between Baseline e-Cigarette Use and Subsequent Cannabis Use Among Baseline Never Cannabis Users, With Participants Answering “Don’t Know” or “Refused” Considered Users or Nonusers of the Product **eTable 9.** Association Between Baseline e-Cigarette Use and Subsequent Past 12-Month Cannabis Vaping Among Baseline Never Cannabis Users **eTable 10.** Predicted Changes in Youth Cannabis Use (2018-2019) Due to Changes in e-Cigarette Use (2017-2018), Assuming Estimated Association to be 100% Causal
