# Supplementary material for: Use of Electronic Cigarettes Among Cannabis-Naive Adolescents and Its Association With Future Cannabis Use
Source: JAMA Netw Open. 2022 Jul 22;5(7):e2223277. doi: 10.1001/jamanetworkopen.2022.23277 (PMC9308048; doi:10.1001/jamanetworkopen.2022.23277)
Supplement: Supplement. — eFigure. Flowchart for Sample Construction eTable 1. Association Between Baseline e-Cigarette Use and Subsequent Cannabis Use (Past 12-Month and Past 30-Day) Among Baseline Never Cannabis Users, Comparing aRRs and aORs eTable 2. Association Between Baseline Ever e-Cigarette Use and Subsequent Cannabis Use (Past 12-Month and Past 30-Day) Among Baseline Never Cannabis Users eTable 3. Association Between Baseline Past 12-Month e-Cigarette Use and Subsequent Cannabis Use (Past 12-Month and Past 30-Day) Among Baseline Never Cannabis Users eTable 4. Association Between Baseline Past 30-Day e-Cigarette Use and Subsequent Cannabis Use (Past 12-Month and Past 30-Day) Among Baseline Never Cannabis Users eTable 5. Global Appraisal of Individual Needs – Short Screener (GAIN-SS) Items eTable 6. Association Between Baseline e-Cigarette Use and Subsequent Cannabis Use Among Baseline Never Cannabis Users, With Additional Measures of Internalizing and Externalizing Problems eTable 7. Association Between Baseline e-Cigarette Use and Subsequent Cannabis Use Among Baseline Never Cannabis Users, Without Sensation Seeking eTable 8. Association Between Baseline e-Cigarette Use and Subsequent Cannabis Use Among Baseline Never Cannabis Users, With Participants Answering “Don’t Know” or “Refused” Considered Users or Nonusers of the Product eTable 9. Association Between Baseline e-Cigarette Use and Subsequent Past 12-Month Cannabis Vaping Among Baseline Never Cannabis Users eTable 10. Predicted Changes in Youth Cannabis Use (2018-2019) Due to Changes in e-Cigarette Use (2017-2018), Assuming Estimated Association to be 100% Causal [file jamanetwopen-e2223277-s001.pdf]

Supplementary Online Content

Sun R, Mendez D, Warner KE. Use of electronic cigarettes among cannabis-naive adolescents and its association with future cannabis use. *JAMA Netw Open.* 2022;5(7):e2223277. doi:10.1001/jamanetworkopen.2022.23277

eFigure. Flowchart for Sample Construction
eTable 1. Association Between Baseline e-Cigarette Use and Subsequent Cannabis Use (Past 12-Month and Past 30-Day) Among Baseline Never Cannabis Users, Comparing aRRs and aORs
eTable 2. Association Between Baseline Ever e-Cigarette Use and Subsequent Cannabis Use (Past 12-Month and Past 30-Day) Among Baseline Never Cannabis Users
eTable 3. Association Between Baseline Past 12-Month e-Cigarette Use and Subsequent Cannabis Use (Past 12-Month and Past 30-Day) Among Baseline Never Cannabis Users
eTable 4. Association Between Baseline Past 30-Day e-Cigarette Use and Subsequent Cannabis Use (Past 12-Month and Past 30-Day) Among Baseline Never Cannabis Users
eTable 5. Global Appraisal of Individual Needs – Short Screener (GAIN-SS) Items
eTable 6. Association Between Baseline e-Cigarette Use and Subsequent Cannabis Use Among Baseline Never Cannabis Users, With Additional Measures of Internalizing and Externalizing Problems
eTable 7. Association Between Baseline e-Cigarette Use and Subsequent Cannabis Use Among Baseline Never Cannabis Users, Without Sensation Seeking
eTable 8. Association Between Baseline e-Cigarette Use and Subsequent Cannabis Use Among Baseline Never Cannabis Users, With Participants Answering "Don't Know" or "Refused" Considered Users or Nonusers of the Product
eTable 9. Association Between Baseline e-Cigarette Use and Subsequent Past 12-Month Cannabis Vaping Among Baseline Never Cannabis Users
eTable 10. Predicted Changes in Youth Cannabis Use (2018-2019) Due to Changes in e-Cigarette Use (2017-2018), Assuming Estimated Association to be 100% Causal

This supplementary material has been provided by the authors to give readers additional information about their work.

© 2022 Sun R et al. *JAMA Network Open.*

eFigure. Flowchart for Sample Construction

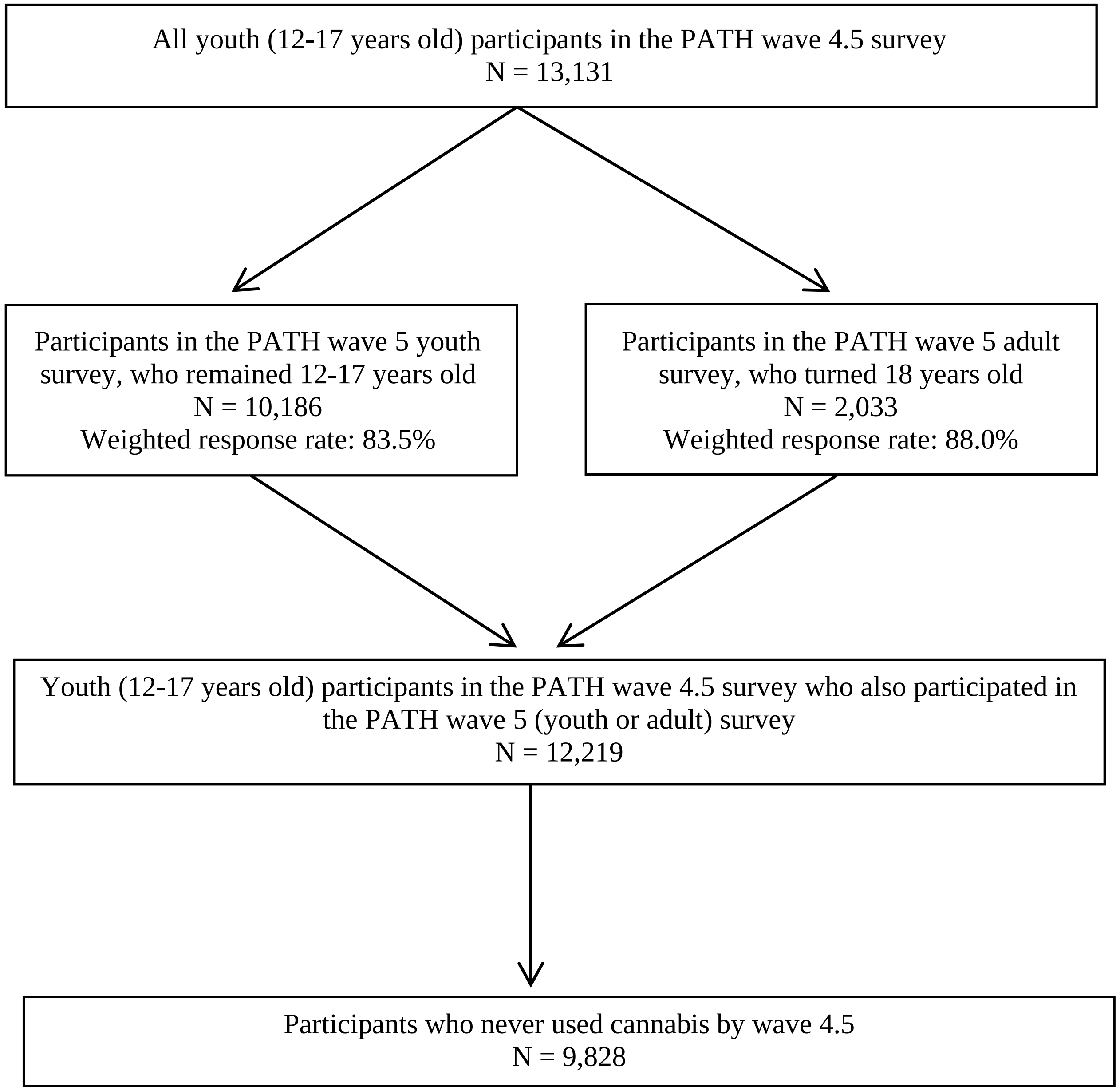

© 2022 Sun R et al. *JAMA Network Open.*

eTable 1. Association Between Baseline e-Cigarette Use and Subsequent Cannabis Use (Past 12-Month and Past 30-Day) Among Baseline Never Cannabis Users, Comparing aRRs and aORs

Wave 4.5 e-cigarettes use	Past 12-month cannabis use		Past 30-day cannabis use	
	aRR[a] (95% CI)	aOR[b] (95% CI)	aRR[a] (95% CI)	aOR[b] (95% CI)
Ever e-cig use	2.57 (2.04-3.09)	3.40 (2.59-4.46)	3.20 (2.10-4.31)	3.66 (2.47-5.42)
Past 12-month e-cig use	2.62 (2.10-3.15)	3.56 (2.70-4.71)	3.40 (2.17-4.63)	3.96 (2.59-6.04)
Past 30-day e-cig use	2.18 (1.50-2.85)	2.84 (1.82-4.44)	2.96 (1.52-4.40)	3.46 (1.92-6.26)

[a] Adjusted relative risk. Adjusted for all study covariates: age, sex, race/ethnicity, highest parental education, household income, school grades, family tobacco use, peer tobacco use, ever tobacco product use, past 12-month alcohol use, ever nonmedical prescription drug use, and sensation seeking.
[b] Adjusted odds ratio. Adjusted for all study covariates, same as listed above.

© 2022 Sun R et al. *JAMA Network Open.*

eTable 2. Association Between Baseline Ever e-Cigarette Use and Subsequent Cannabis Use (Past 12-Month and Past 30-Day) Among Baseline Never Cannabis Users

Wave 4.5 variables	Past 12-month cannabis use in wave 5		Past 30-day cannabis use in wave 5	
	Coefficient (95% CI)	P	Coefficient (95% CI)	P
Ever e-cig use	**1.22 (.95,1.49)**	**<.001**	**1.30 (0.90,1.69)**	**<.001**
Age (REF: 12-14)				
15-17	**0.53 (0.33,0.74)**	**<.001**	**0.58 (0.26,0.91)**	**.001**
Female	0.16 (-0.03,0.36)	.09	0.15 (-0.11,0.41)	.25
Race/ethnicity (REF: NH white)				
NH black	**0.66 (0.40,0.92)**	**<.001**	**0.55 (0.14,0.96)**	**.009**
Hispanic	**0.36 (0.08,0.63)**	**.01**	0.23 (-0.11,0.56)	.19
NH other	0.07 (-0.25,0.40)	.67	-0.05 (-0.58,0.48)	.86
Highest parental education (REF: ≤ High school/ GED)				
Some college	-0.18 (-0.49,0.13)	.25	-0.33 (-0.68,0.03)	.07
≥ College	-0.18 (-0.55,0.20)	.35	-0.24 (-0.66,0.17)	.25
Household income (REF: < 50k)				
50k to 100k	0.01 (-0.26,0.27)	.97	0.08 (-0.26,0.41)	.65
> 100k	0.18 (-0.15,0.50)	.28	0.35 (-0.08,0.78)	.11
School grades (REF: < mostly B's)				
≥ *mostly B's*	**-0.38 (-0.60,-0.17)**	**.001**	-0.16 (-0.51,0.20)	.39
Family tobacco use	**0.37 (0.16,0.57)**	**.001**	**0.32 (0.06,0.58)**	**.02**
Peer tobacco use	**0.63 (0.44,0.82)**	**<.001**	**0.54 (0.24,0.84)**	**.001**
Ever tobacco product use (excluding e-cigs)	**0.54 (0.18,0.89)**	**.003**	0.30 (-0.12,0.72)	.16
Past 12-month alcohol use	**0.83 (0.57,1.08)**	**<.001**	0.34 (-0.002,0.67)	.05
Ever nonmedical prescription drug use	0.23 (-0.01,0.47)	.07	0.23 (-0.09,0.55)	.16
Sensation seeking[a]	**0.24 (0.15,0.33)**	**<.001**	**0.29 (0.15,0.42)**	**<.001**
Sample Size (N)[b]	7,011		7,032	

Notes

[a] Corresponds to 1 unit increase in the risk of sensation seeking (5-point scale).

[b] Effective sample size; participants with missing data were excluded.

Figures bolded if p < .05.

© 2022 Sun R et al. *JAMA Network Open.*

eTable 3. Association Between Baseline Past 12-Month e-Cigarette Use and Subsequent Cannabis Use (Past 12-Month and Past 30-Day) Among Baseline Never Cannabis Users

Wave 4.5 variables	Past 12-month cannabis use in wave 5		Past 30-day cannabis use in wave 5	
	Coefficient (95% CI)	P	Coefficient (95% CI)	P
Past 12-month e-cig use	**1.27 (0.99,1.55)**	**<.001**	**1.38 (0.95,1.80)**	**<.001**
Age (REF: 12-14)				
15-17	**0.57 (0.37,0.76)**	**<.001**	**0.62 (0.30,0.94)**	**<.001**
Female	0.16 (-0.03,0.35)	.10	0.14 (-0.12,0.39)	.28
Race/ethnicity (REF: NH white)				
NH black	**0.62 (0.36,0.89)**	**<.001**	**0.52 (0.12,0.92)**	**.01**
Hispanic	**0.37 (0.10,0.64)**	**.008**	0.25 (-0.10,0.59)	.16
NH other	0.08 (-0.24,0.40)	.63	-0.03 (-0.55,0.50)	.92
Highest parental education (REF: ≤ High school/ GED)				
Some college	-0.19 (-0.50,0.11)	.22	-0.35 (-0.71,0.01)	.06
≥ College	-0.20 (-0.57,0.17)	.29	-0.28 (-0.69,0.13)	.18
Household income (REF: < 50k)				
50k to 100k	-0.02 (-0.28,0.25)	.91	0.05 (-0.28,0.39)	.75
> 100k	0.16 (-0.15,0.48)	.31	0.33 (-0.08,0.75)	.12
School grades (REF: < mostly B's)				
≥ *mostly B's*	**-0.40 (-0.62,-0.18)**	**.001**	-0.17 (-0.53,0.18)	.34
Family tobacco use	**0.39 (0.18,0.59)**	**<.001**	**0.34 (0.08,0.60)**	**.01**
Peer tobacco use	**0.61 (0.42,0.80)**	**<.001**	**0.51 (0.20,0.81)**	**.001**
Ever tobacco product use (excluding e-cigs)	**0.63 (0.27,1.00)**	**.001**	0.38 (-0.07,0.82)	.10
Past 12-month alcohol use	**0.83 (0.57,1.08)**	**<.001**	0.32 (-0.02,0.66)	.06
Ever nonmedical prescription drug use	**0.25 (0.02,0.49)**	**.03**	0.26 (-0.05,0.57)	.10
Sensation seeking[a]	**0.24 (0.15,0.34)**	**<.001**	**0.29 (0.16,0.43)**	**<.001**
Sample Size (N)[b]	7,018		7,039	

Notes
[a] Corresponds to 1 unit increase in the risk of sensation seeking (5-point scale).
[b] Effective sample size; participants with missing data were excluded.
Figures bolded if p < .05.

© 2022 Sun R et al. *JAMA Network Open.*

eTable 4. Association Between Baseline Past 30-Day e-Cigarette Use and Subsequent Cannabis Use (Past 12-Month and Past 30-Day) Among Baseline Never Cannabis Users

Wave 4.5 variables	Past 12-month cannabis use in wave 5		Past 30-day cannabis use in wave 5	
	Coefficient (95% CI)	P	Coefficient (95% CI)	P
Past 30-day e-cig use	**1.04 (0.60,1.49)**	**<.001**	**1.24 (0.65,1.83)**	**<.001**
Age (REF: 12-14)				
15-17	**0.62 (0.42,0.81)**	**<.001**	**0.68 (0.36,1.00)**	**<.001**
Female	0.16 (-0.03,0.36)	.09	0.14 (-0.12,0.40)	.30
Race/ethnicity (REF: NH white)				
NH black	**0.56 (0.30,0.82)**	**<.001**	**0.45 (0.06,0.84)**	**.02**
Hispanic	**0.33 (0.07,0.60)**	**.02**	0.21 (-0.14,0.55)	.23
NH other	0.04 (-0.29,0.36)	.83	-0.07 (-0.59,0.46)	.81
Highest parental education (REF: ≤ High school/ GED)				
Some college	-0.20 (-0.50,0.10)	.19	**-0.36 (-0.72,-0.01)**	**.05**
≥ College	-0.18 (-0.54,0.18)	.33	-0.26 (-0.66,0.15)	.21
Household income (REF: < 50k)				
50k to 100k	-0.02 (-0.28,0.24)	.87	0.04 (-0.30,0.38)	.81
> 100k	0.17 (-0.14,0.47)	.29	0.33 (-0.08,0.74)	.12
School grades (REF: < mostly B's)				
≥ *mostly B's*	**-0.41 (-0.63,-0.20)**	**<.001**	-0.19 (-0.55,0.16)	.28
Family tobacco use	**0.40 (0.20,0.60)**	**<.001**	**0.36 (0.10,0.63)**	**.008**
Peer tobacco use	**0.71 (0.53,0.90)**	**<.001**	**0.62 (0.34,0.91)**	**<.001**
Ever tobacco product use (excluding e-cigs)	**0.76 (0.40,1.11)**	**<.001**	**0.48 (0.03,0.93)**	**.04**
Past 12-month alcohol use	**0.91 (0.66,1.16)**	**<.001**	**0.46 (0.13,0.78)**	**.01**
Ever nonmedical prescription drug use	**0.26 (0.03,0.49)**	**.02**	0.26 (-0.04,0.57)	.09
Sensation seeking[a]	**0.25 (0.16,0.34)**	**<.001**	**0.30 (0.17,0.43)**	**<.001**
Sample Size (N)[b]	7,017		7,038	

Notes

[a] Corresponds to 1 unit increase in the risk of sensation seeking (5-point scale).

[b] Effective sample size; participants with missing data were excluded.

Figures bolded if p < .05.

© 2022 Sun R et al. *JAMA Network Open.*

eTable 5. Global Appraisal of Individual Needs – Short Screener (GAIN-SS) Items

Internalizing Problem Symptoms	1) Feeling very trapped, lonely, sad, blue, depressed or hopeless about the future
	2) Sleep trouble – such as bad dreams, sleeping restlessly or falling asleep during the day
	3) Feeling very anxious, nervous, tense, scared, panicked or like something bad was going to happen
	4) Becoming very distressed and upset when something reminded you of the past
Externalizing Problem Symptoms	1) Lied or conned to get things you wanted or to avoid having to do something
	2) Had a hard time paying attention at school, work or home
	3) Had a hard time listening to instructions at school, work or home
	4) Were a bully or threatened other people
	5) Started physical fights with other people
	6) Felt restless or the need to run around or climb on things
	7) Gave answers before the other person finished asking the question

Notes

Severity of internalizing problem in the past 12 months: 0-1 symptoms (low), 2-3 symptoms (moderate), 4 symptoms (high).

Severity of externalizing problem in the past 12 months: 0-1 symptoms (low), 2-3 symptoms (moderate), ≥4 symptoms (high).

High internalizing problem scores are closely associated with disorders such as depression, anxiety, trauma, and schizophrenia and bipolar disorder. High externalizing problem scores are closely associated with diagnoses of attention deficit disorders and impulsivity.

© 2022 Sun R et al. *JAMA Network Open.*

eTable 6. Association Between Baseline e-Cigarette Use and Subsequent Cannabis Use Among Baseline Never Cannabis Users, With Additional Measures of Internalizing and Externalizing Problems

Wave 4.5 e-cigarettes use	Cannabis use in wave 5				
	aRR[a] (95% CI)	aRD[b] (95% CI)	Risk w/o e-cig use % (95% CI)	Risk w/ e-cig use % (95% CI)	Sample Size[c] N
	Past 12-Month Use				
Ever e-cig use	2.57 (2.04-3.10)	13.98 (9.83-18.13)	8.90 (8.04-9.75)	22.87 (18.92-26.83)	7,011
Past 12-month e-cig use	2.63 (2.09-3.16)	14.91 (10.50-19.33)	9.17 (8.33-10.00)	24.08 (19.81-28.35)	7,018
Past 30-day e-cig use	2.17 (1.50-2.84)	11.78 (5.29-18.27)	10.07 (9.24-10.91)	21.82 (15.57-28.14)	7,017
	Past 30-Day Use				
Ever e-cig use	3.22 (2.12-4.31)	7.99 (4.55-11.43)	3.61 (3.06-4.16)	11.60 (8.29-14.91)	7,032
Past 12-month e-cig use	3.41 (2.20-4.62)	8.97 (4.86-13.07)	3.72 (3.20-4.24)	12.69 (8.67-16.71)	7,039
Past 30-day e-cig use	2.95 (1.52-4.37)	8.23 (2.31-14.16)	4.23 (3.73-4.74)	12.46 (6.56-18.37)	7,038

Notes

[a] Adjusted relative risk. Adjusted for all study covariates: age, sex, race/ethnicity, highest parental education, household income, school grades, family tobacco use, peer tobacco use, ever tobacco product use (excluding e-cigarettes), past 12-month alcohol use, ever nonmedical prescription drug use, past 12-month internalizing problem (low vs moderate vs high), past 12-month externalizing problem (low vs moderate vs high), and sensation seeking.

[b] Adjusted risk difference, in percentage points. Adjusted for all study covariates, same as listed above.

[c] Effective sample size; participants with missing data were excluded.

eTable 7. Association Between Baseline e-Cigarette Use and Subsequent Cannabis Use Among Baseline Never Cannabis Users, Without Sensation Seeking

Wave 4.5 e-cigarettes use	Cannabis use in wave 5				
	aRR[a] (95% CI)	aRD[b] (95% CI)	Risk w/o e-cig use % (95% CI)	Risk w/ e-cig use % (95% CI)	Sample Size[c] N
	Past 12-Month Use				
Ever e-cig use	2.54 (2.08-3.00)	13.93 (10.19-17.67)	9.06 (8.28-9.84)	22.99 (19.39-26.59)	8,170
Past 12-month e-cig use	2.60 (2.11-3.09)	14.89 (10.72-19.06)	9.32 (8.55-10.09)	24.21 (20.17-28.25)	8,182
Past 30-day e-cig use	2.24 (1.64-2.85)	12.64 (6.70-18.58)	10.19 (9.42-10.95)	22.83 (17.01-28.64)	8,181
	Past 30-Day Use				
Ever e-cig use	3.11 (2.19-4.02)	7.75 (4.78-10.73)	3.68 (3.19-4.17)	11.43 (8.56-14.31)	8,197
Past 12-month e-cig use	3.31 (2.22-4.41)	8.75 (4.95-12.55)	3.78 (3.31-4.25)	12.54 (8.82-16.25)	8,209
Past 30-day e-cig use	2.94 (1.69-4.20)	8.30 (3.10-13.50)	4.27 (3.79-4.75)	12.57 (7.43-17.71)	8,208

Notes

[a] Adjusted relative risk. Adjusted for all study covariates: age, sex, race/ethnicity, highest parental education, household income, school grades, family tobacco use, peer tobacco use, ever tobacco product use (excluding e-cigarettes), past 12-month alcohol use, and ever nonmedical prescription drug use.

[b] Adjusted risk difference, in percentage points. Adjusted for all study covariates, same as listed above.

[c] Effective sample size; participants with missing data were excluded.

© 2022 Sun R et al. *JAMA Network Open.*

eTable 8. Association Between Baseline e-Cigarette Use and Subsequent Cannabis Use Among Baseline Never Cannabis Users, With Participants Answering "Don't Know" or "Refused" Considered Users or Nonusers of the Product

Wave 4.5 e-cigarettes use	Cannabis use in wave 5				
	aRR[a] (95% CI)	aRD[b] (95% CI)	Risk w/o e-cig use % (95% CI)	Risk w/ e-cig use % (95% CI)	Sample Size[c] N
	**Past 12-Month Use**				
	User[d]				
Ever e-cig use	2.56 (2.05-3.08)	13.97 (9.87-18.07)	8.94 (8.11-9.78)	22.92 (19.00-26.83)	7,117
Past 12-month e-cig use	2.63 (2.11-3.14)	14.99 (10.64-19.33)	9.22 (8.41-10.03)	24.20 (19.98-28.43)	7,125
Past 30-day e-cig use	2.16 (1.49-2.83)	11.73 (5.23-18.23)	10.12 (9.30-10.93)	21.85 (15.53-28.16)	7,124
	Nonuser[e]				
Ever e-cig use	2.57 (2.06-3.08)	14.21 (10.13-18.29)	9.04 (8.21-9.86)	23.25 (19.35-27.14)	7,129
Past 12-month e-cig use	2.62 (2.12-3.13)	15.15 (10.81-19.48)	9.32 (8.53-10.12)	24.47 (20.26-28.69)	7,137
Past 30-day e-cig use	2.16 (1.50-2.83)	11.92 (5.38-18.45)	10.23 (9.42-11.04)	22.15 (15.79-28.50)	7,136
	**Past 30-Day Use**				
	User[d]				
Ever e-cig use	3.24 (2.15-4.34)	8.05 (4.62-11.48)	3.58 (3.05-4.11)	11.63 (8.34-14.93)	7,141
Past 12-month e-cig use	3.43 (2.20-4.65)	9.00 (4.85-13.14)	3.70 (3.20-4.21)	12.70 (8.65-16.75)	7,149
Past 30-day e-cig use	2.97 (1.53-4.41)	8.30 (2.32-14.27)	4.21 (3.72-4.69)	12.50 (6.55-18.46)	7,148
	Nonuser[e]				
Ever e-cig use	3.21 (2.16-4.27)	8.05 (4.69-11.42)	3.64 (3.12-4.16)	11.69 (8.46-14.93)	7,202
Past 12-month e-cig use	3.44 (2.24-4.64)	9.14 (5.04-13.25)	3.75 (3.26-4.24)	12.89 (8.88-16.91)	7,211

© 2022 Sun R et al. *JAMA Network Open.*

Past 30-day e-cig use	2.99 (1.58-4.41)	8.49 (2.59-14.39)	4.26 (3.78-4.74)	12.75 (6.89-18.61)	7,210

Notes

[a] Adjusted relative risk. Adjusted for all study covariates: age, sex, race/ethnicity, highest parental education, household income, school grades, family tobacco use, peer tobacco use, ever tobacco product use (excluding e-cigarettes), past 12-month alcohol use, ever nonmedical prescription drug use, and sensation seeking.

[b] Adjusted risk difference, in percentage points. Adjusted for all study covariates, same as listed above.

[c] Effective sample size; participants with missing data were excluded.

[d] Participants who answered "don't know" or "refused" are considered users of that product.

[e] Participants who answered "don't know" or "refused" are considered nonusers of that product.

© 2022 Sun R et al. *JAMA Network Open.*

eTable 9. Association Between Baseline e-Cigarette Use and Subsequent Past 12-Month Cannabis Vaping Among Baseline Never Cannabis Users

**Wave 4.5 e-cigarettes use**	**Past 12-month cannabis vaping in wave 5**				
	aRR[a] (95% CI)	aRD[b] (95% CI)	Risk w/o e-cig use % (95% CI)	Risk w/ e-cig use % (95% CI)	Sample Size[c]
Ever e-cig use	2.64 (1.98-3.30)	9.42 (6.16-12.68)	5.74 (5.02-6.45)	15.15 (12.08-18.22)	7,032
Past 12-month e-cig use	2.72 (2.06-3.39)	10.18 (6.77-13.59)	5.91 (5.21-6.61)	16.09 (12.84-19.34)	7,039
Past 30-day e-cig use	1.92 (1.25-2.59)	6.17 (1.92-10.41)	6.68 (5.97-7.38)	12.84 (8.82-16.87)	7,038

[a] Adjusted relative risk. Adjusted for all study covariates: age, sex, race/ethnicity, highest parental education, household income, school grades, family tobacco use, peer tobacco use, ever tobacco product use (excluding e-cigarettes), past 12-month alcohol use, ever nonmedical prescription drug use, and sensation seeking.
[b] Adjusted odds ratio. Adjusted for all study covariates, same as listed above.
[c] Effective sample size; participants with missing data were excluded.

© 2022 Sun R et al. *JAMA Network Open.*

eTable 10. Predicted Changes in Youth Cannabis Use (2018-2019) Due to Changes in e-Cigarette Use (2017-2018), Assuming Estimated Association to be 100% Causal

	Cannabis Use			
E-cigarette use	Estimated Association (aRD[a])		Predicted Changes[b] (in percentage points)	
	Past 12-month	Past 30-day	Past 12-month	Past 30-day
Ever use	13.93	7.96	0.57	0.33
Past 30-day use	11.86	8.29	0.80	0.68

Notes
Youth past 30-day use of e-cigarettes grew 5.73 percentage points (8.06% to 13.79%) and ever use of e-cigarettes grew 4.09 percentage points (21.39% to 25.48%) from 2017 to 2018, using National Youth Tobacco Survey.
[a] Adjusted risk difference, in percentage points.
[b] Predicted changes (percentage points) are calculated as: changes in e-cigarette use (percentage points) x estimated association (percentage points) / 100.

© 2022 Sun R et al. *JAMA Network Open.*
